# Do Bilinguals Outperform Monolinguals in Switching Tasks? Contrary Evidence for Nonlinguistic and Linguistic Switching Tasks

**DOI:** 10.1162/nol_a_00059

**Published:** 2021-12-23

**Authors:** Ernest Mas-Herrero, Daniel Adrover-Roig, María Ruz, Ruth de Diego-Balaguer

**Affiliations:** Cognition and Brain Plasticity Unit, Bellvitge Biomedical Research Institute [IDIBELL], L’Hospitalet de Llobregat, Barcelona, Spain; Department of Cognition, Development and Education Psychology, University of Barcelona, Barcelona, Spain; Institute of Neurosciences, University of Barcelona, Barcelona, Spain; Department of Applied Pedagogy and Educational Psychology, Institute of Research and Innovation in Education (IRIE), University of Balearic Islands, Palma, Spain; Mind, Brain and Behavior Research Center (CIMCYC). Department of Experimental Psychology. University of Granada, Granada, Spain; Institució Catalana de Recerca i Estudis Avançats, Barcelona, Spain

**Keywords:** bilingualism, cognitive control, executive function, task-switching, switching cost

## Abstract

The benefits of bilingualism in executive functions are highly debated. Even so, in switching tasks, these effects seem robust, although smaller than initially thought (Gunnerud et al., 2020; Ware et al., 2020). By handling two languages throughout their lifespan, bilinguals appear to train their executive functions and show benefits in nonlinguistic switching tasks compared to monolinguals. Nevertheless, because bilinguals need to control for the interference of another language, they may show a disadvantage when dealing with task-switching paradigms requiring language control, particularly when those are performed in their less dominant language. The present work explored this issue by studying bilingualism’s effects on task switching within the visual and language domains. On the one hand, our results show that bilinguals were overall faster and presented reduced switch costs compared to monolinguals when performing perceptual geometric judgments with no time for task preparation. On the other hand, no bilingual advantage was found when a new sample of comparable bilinguals and monolinguals completed a within-language switching task. Our results provide clear evidence favoring the bilingual advantage, yet only when the task imposes greater executive demands and does not involve language control.

## INTRODUCTION

The impact of bilingualism on executive functions is an exciting but highly debated research topic in psychology and cognitive neuroscience. Several studies suggest that both languages of a bilingual are active during perception and production even when only one language is used (Colomé, 2001; Rodriguez-Fornells et al., 2006; Thierry & Wu, 2007; van Heuven et al., 2008), generating a constant conflict between the two languages (Green & Abutalebi, 2013). However, bilinguals efficiently handle both languages and show a remarkable ability to switch between them (language switching) without apparent effort. This is particularly noticeable in language-mixing environments where both languages are highly used.

Neuroimaging studies have revealed that both language monitoring and switching engage key brain regions of cognitive control such as the anterior cingulate cortex, the inferior frontal gyrus, the basal ganglia, and parietal areas (Abutalebi & Green, 2007; Garbin et al., 2010; Rodriguez-Fornells et al., 2006; van Heuven et al., 2008). The fact that language control in bilinguals shares, at least partially, functional and neural mechanisms with domain-general executive control processes has led to the idea that the bilingual brain may rely on executive functions to avoid interference from the language not currently in use and to switch between languages (Green & Abutalebi, 2013). Given the impact of experience on cognitive performance (e.g., Bengtsson et al., 2005), it has been proposed that the extensive use of executive functions in language control may improve these functions outside the language domain, leading to a bilingual advantage over monolinguals (Barac & Bialystok, 2012; Bialystok, 1999; Christoffels et al., 2013; Kroll & Chiarello, 2016; Luk et al., 2010; Mechelli et al., 2004; Wiseheart et al., 2016). In this vein, results suggest that even a short language switching training can reduce switch costs in nonlinguistic tasks, revealing the transfer of training effects from linguistic to nonlinguistic domains (Timmer et al., 2019).

However, the size and even the existence of the “bilingual advantage” is still a matter of fervid debate (Dick et al., 2019; Paap et al., 2014, 2020; Sanchez-Azanza et al., 2017). Recent meta-analyses suggest that such advantage is, in fact, smaller than initially thought, and it is not an overall effect on executive functions but rather depends on the type of executive function studied. Concretely, one of the most consistent and reliable bilingual advantages through the lifespan is a reduced switching cost in nonlinguistic task-switching paradigms (Gunnerud et al., 2020; Ware et al., 2020). Switching costs are usually measured as the difference between *switch* and *non-switch* trials in blocks where tasks randomly vary (Monsell, 2003). Responses take longer to initiate on a switch than on a *stay* trial, reflecting the extra processing demands associated with reconfiguring the task sets. Notably, the bilingual advantage in cognitive control is generally observed in the most demanding conditions, where higher cognitive control needs to be deployed (Bialystok et al., 2006; Costa et al., 2009; Hernández et al., 2013).

Nevertheless, it is worth noting that bilinguals, as opposed to monolinguals, use cognitive control even in monolingual contexts, where only one language is needed, in order to maintain the language in use and avoid interferences from the other language (Abutalebi & Green, 2016; Wodniecka et al., 2021). This implies that bilinguals should have their cognitive control abilities taxed in contexts demanding language control (i) compared to monolinguals—even when using only one language, and (ii) compared to bilinguals’ abilities in nonlinguistic contexts. In other words, does the bilinguals’ constant need for language control affect their ongoing general executive functions performance when simultaneously dealing with language selection? In this sense, it is worth questioning whether the bilingual advantage in task-switching paradigms is still present when switching needs to be performed in a context in which language control is also demanded. This question is particularly pertinent given that language is omnipresent in our lives. If language monitoring involves cognitive resources, this has significant consequences when considering bilingualism’s benefits as well as disadvantages. Whereas the bilinguals’ training of executive functions may benefit nonlinguistic task switching, the bilinguals’ unavoidable and continuous need for executive resources to select the appropriate language may worsen their performance in task switching when simultaneously dealing with language selection. For instance, switching between linguistic tasks (e.g., phonetic and semantic judgments) within one language may become more demanding for bilinguals. Unlike monolinguals, they need to continuously overcome the interference from the unused language in addition to engaging executive control to switch between task sets. This effect may be particularly evident when performing tasks in the second language.

To address this question, we tested two independent but comparable samples of Spanish monolinguals and Spanish/Catalan bilinguals in two distinct task-switching paradigms with or without language control demands. Given the evidence that the bilingualism advantage is mainly observed under tasks involving high cognitive control demands (e.g., Costa et al., 2009; Hernández et al., 2013), both tasks included two conditions that differed in their cognitive demands. In the low-cognitive demand condition, cues indicating which task to perform were presented before each target so participants could focus their attention in advance on the information relevant for the upcoming task (Easy block). In the high-cognitive demand condition, task information was only conveyed by the target color, which prevented preparation and required rapid task-set reconfiguration (Hard block). The first is associated with smaller switching costs than the latter due to preparatory effects (Monsell, 2003). We used this paradigm in two experiments. In Experiment 1, bilinguals and monolinguals performed geometric judgments (orientation or shape), and in Experiment 2, participants carried out linguistic (phonological or semantic) judgments on Spanish words. Each experiment consisted of two independent but comparable samples of Spanish/Catalan bilinguals and Spanish monolinguals. Whereas the first experiment compared switch costs outside the language domain, the second one explored whether bilinguals’ potential advantage remains if language control is required. Besides, in the second experiment, two groups of bilinguals were included, one group of sequential bilinguals that learned Spanish later than Catalan and one group of simultaneous bilinguals that acquired Spanish and Catalan concurrently.

We hypothesized that bilinguals would present a reduced switch-cost compared to monolinguals for the nonlinguistic switching task in Experiment 1, particularly in the most demanding condition, as previously reported. However, if performing the task with linguistic processing incurs an additional cost for bilinguals, these differences should vanish or even favor monolinguals in the within-language task from Experiment 2. If so, we would also expect bilinguals’ performance to be associated with their language dominance. Concretely, since the within-language task-switching paradigm was conducted in Spanish, we hypothesize that those with Catalan dominance would perform worse among bilinguals since they may need more cognitive resources to inhibit their dominant, non-target language (Catalan)—and consequently, may incur a greater cost.

## EXPERIMENT 1 MATERIALS AND METHODS

### Participants

One hundred and ten volunteers participated in this study: 55 Catalan–Spanish bilinguals (mean age = 21.3, *SD* = 4.16; 43 women) and 55 monolingual participants (mean age = 20.6, *SD* = 2.3; 45 women). Six participants from the bilingual group and eight from the monolingual group were not included in the analyses because they had more than 30% error rates in at least one of the blocks. Bilinguals were born in Catalonia (in the North-East of Spain), where they were tested. They had received a bilingual Spanish–Catalan education and were fully fluent in the two languages, in both listening and reading skills. They also used both languages during their daily life and were early bilinguals beginning their exposure to their L2 at ages ranging from birth to 3.5 years (Table 1). Indeed, all Catalan students are Spanish–Catalan bilinguals and experience a similar degree of exposure to both languages. For instance, schooling begins at 3 years of age, and the language used at school is Catalan. However, Spanish is most present in the media (e.g., TV, cinema, and books). To access the university, they all need to pass the Spanish University Access Tests that include a Spanish language exam (as the rest of Spain, and of comparable difficulty) and, in addition, a Catalan language exam. Therefore, they are all highly fluent and familiar with both languages.

**Table 1.** Language history, self-evaluated proficiency scores, and BSWQ scores for participants in Experiment 1.

	**Bilinguals**		**Monolinguals**
*N*	49		47
Age	21.3 (0.6)		20.6 (0.3)
*Language History Questionnaire*			
Use of language	3.23 (0.16)		
*Proficiency scores*	*Spanish*	*Catalan*	
Age of acquisition	2.9 (0.2)	2.7 (0.2)	
Comprehension	3.96 (0.03)	3.96 (0.03)	
Reading	3.69 (0.06)	3.65 (0.07)	
Production	3.98 (0.02)	3.98 (0.02)	
Writing	3.73 (0.06)	3.76 (0.06)	
Total	3.84 (0.04)	3.84 (0.03)	
*BSWQ* scores			
Sp_Cat	7.37 (0.27)		
Cat_Sp	8.70 (0.36)		
Contextual	8.02 (0.34)		
Unconscious	6.70 (0.35)		
Overall	30.78 (0.91)		

*Note*. Age and scores are reported as mean (standard error of the mean). BSWQ = Bilingual Switching Questionnaire. Use of language refers to the self-evaluation rates used in a 7-point scale ranging from 1 = *Catalan only*; 2 = *Catalan frequently, Spanish rarely*; 3 = *Catalan majority with Spanish at least 1/4 of the time*; 4 = *Equal use of Spanish and Catalan*; 5 = *Spanish majority with Catalan at least 1/4 of the time*; 6 = *Spanish frequently; Catalan rarely*; 7 = *Spanish only*. For use of language, 51% of the participants provided self-ratings between 3 and 5, 43% ratings below 3, and 6% ratings above 5. Proficiency scores refer to the self-evaluation rates in a 4-point scale ranging from 4 = *native speaker level* to 1 = *complete ignorance of the language*. Sp_Cat = Tendencies to switch from Catalan to Spanish; Cat_Sp = Tendencies to switch from Spanish to Catalan.

On the other hand, monolinguals were born in Andalusia (in the South of Spain), where they were tested. Monolingual participants were educated only in Spanish except for the English lessons that all students (bilinguals and monolinguals) received in the schooling courses. They did not have contact with other languages during their daily lives. Indeed, only participants reporting a low frequency of English use (or other languages, including Catalan in the case of the monolinguals) were selected for the study. In addition, only participants that self-labeled as monolinguals were included in the monolingual group. Bilinguals and monolinguals were all university students and had comparable socio-cultural environments. They were paid cash or course credits for their participation, and they all signed a consent form approved by the local ethics committee.

#### Language questionnaires

The Spanish version of the Language History Questionnaire (LHQ; de Diego-Balaguer et al., 2005; Weber-Fox & Neville, 1996) was administered to all bilingual participants. The LHQ informs about language use in different life stages (kindergarten, school, high school, and as an adult) and scenarios (school, university, home, or others) in a Likert-like item of seven options, adapted to Catalan/Spanish university students, from 1 = *Catalan only*; 2 = *Catalan frequently, Spanish rarely*; 3 = *Catalan majority with Spanish at least 1/4 of the time*; 4 = *Equal use of Spanish and Catalan*; 5 = *Spanish majority with Catalan at least 1/4 of the time*; 6 = *Spanish frequently; Catalan rarely*; 7 = *Spanish only*. The average score was used to measure their Spanish/Catalan dominance (higher values indicate Spanish dominance, lower values, Catalan). We also evaluated the self-assessed proficiency in the oral, written, and comprehension domains for each language using four-point Likert items ranging from 4 = *native speaker level* to 1 = *complete ignorance of the language* (Table 1). Bilingual participants also responded to the Bilingual Switching Questionnaire (BSWQ; Rodriguez-Fornells et al., 2012). The BSWQ included 12 questions representing four subscales: (1) Tendencies to switch from Catalan to Spanish (e.g., “When I cannot find a word in Catalan, I tend to produce it in Spanish right away”), (2) Tendencies to switch from Spanish to Catalan (e.g., “When I cannot find a word in Spanish, I tend to produce it in Catalan right away”), (3) Contextual switches (e.g., “There are situations in which I always switch between languages”), and (4) Unattended switches (e.g., “It is difficult for me to control switching between languages during a conversation”). The answers were given on a 5-point scale.

#### Stimuli and procedure

**Figure 1.**
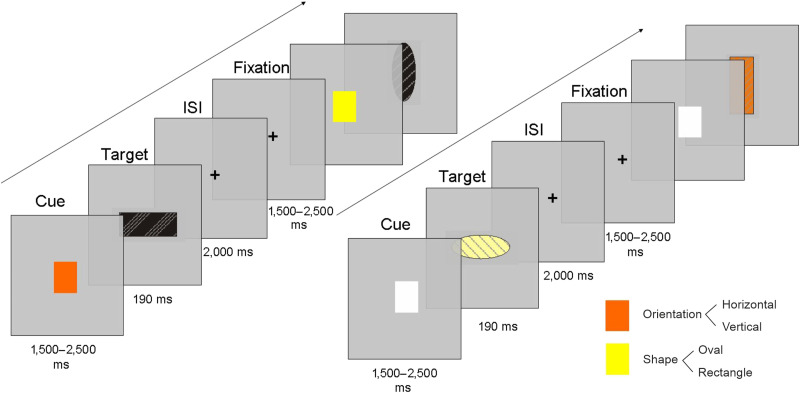
Sequence of events in the two types of blocks used in Experiment 1. Easy blocks included colored squares with black targets (left panel) and Hard blocks included white squares and colored targets (right panel).

We used a nonlinguistic switching paradigm adapted from González-García and colleagues (2016; see Figure 1), which was programmed in E-prime 2.0 (https://pstnet.com/). Participants were asked to classify figures on a trial-by-trial basis, depending on either their shape (ellipse or rectangle) or based on their orientation (horizontal or vertical). The task included two independent experimental blocks, each consisting of 112 trials (for a total of 224 trials). In the Easy block, colored cues (orange or yellow) presented before the targets (displayed in black) informed participants about the task to perform (shape or orientation judgments; color-task associations were counterbalanced across participants). In the Hard block, task information was conveyed by the color (orange or yellow) of the target figures (Figure 1). The order of presentation of both blocks was counterbalanced across participants. Half of the cue stimuli were ellipses, and the other half were rectangles. For each shape, half of the stimuli were in a horizontal, and the other half were in a vertical orientation. Target figures were filled with different lines and points (28 patterns of horizontal, vertical, diagonal, curved lines, and points for a total of 112 different designs) to visually create differentiated figures (and match the number of differentiated stimuli in Experiment 2). In total, 112 different stimuli were repeated twice, once per task. On each block, half of the trials were switch (56 trials), and the other half were non-switch trials (56 trials). Half of the switch (28) and the non-switch trials (28) involved shape judgments, and the other half (28 and 28, respectively), orientation judgments. For each judgment, half of the switch (14) and non-switch trials (14) involved ellipses, and the other half (14 and 14, respectively), rectangles. The order of presentation was randomized within each block and participant. Stimuli size was 2.5 × 1 cm, and they were included in a grey box (3.5 × 1.25 cm), which matched the background display color.

Participants were placed 60 cm in front of a 17” computer screen. They were instructed to respond using the index and middle fingers of their right hand (the assignment of fingers to responses was counterbalanced across participants). Before performing the experimental session, all participants were familiarized with the task by completing a short training session (5 min, 48 trials) with a different set of figures. A grey background remained during the whole task. Each trial started with a fixation cross presented with a random duration from 1,500 ms to 2,500 ms. In the Easy block, the task cue (either yellow or orange) appeared from 1,500 ms to 2,500 ms in the screen center. Afterward, the target figure appeared in black typeface for 190 ms. Targets were presented very rapidly to ensure participants were very attentive to the cue and target during the task. Similar short stimulus durations are frequent in experiments recording electroencephalography (e.g., Ruz & Nobre, 2008), and the 190 ms durations employed were enough for perceiving the figures comfortably. After the target, a fixation cross remained in the center of the screen for 2 s. During this time, participants had to respond (Figure 1). A 100 ms beep immediately followed out-of-time and incorrect responses. In each trial, the cue indicated the specific task to respond.

In the Hard blocks, the sequence of trials was the same except for the colored cues, which were replaced with white uninformative squared stimuli (Figure 1, right panel). Participants were informed about the task to be performed by the color in which the target was displayed (either orange or yellow). The relation between the color and task was counterbalanced across participants and was the same in Easy and Hard blocks for a given participant.

#### Statistical analysis

To test the impact of bilingualism in task-switching performance, we performed linear mixed modeling in R (version 4.0.2) (https://www.npackd.org/p/r/4.0.2) and RStudio (https://www.rstudio.com/) using the lme4 package (Bates, Mächler, et al., 2015). Across all included participants, 21,504 trials were collected. The first trial in a block and trials preceded by an error were excluded from the analysis (10.5% of trials), resulting in 19,235 trials to be analyzed.

Accuracy data were analyzed using generalized linear mixed-effects modeling (using the glmer function). The dependent variable was assumed to have a binomial distribution, and a logit link function was applied. We then generated a model based on a large body of research investigating bilingualism’s impact on cognitive control and task switching in particular. Specifically, based on the results of previous studies showing a bilingual advantage on task switching, and specifically in highly demanding experimental conditions, we included a 3-way interaction between Switching (Non-Switch and Switch), Difficulty (Easy and Hard), and Group (Bilinguals and Monolinguals). To control for differences in performance between tasks, we also included Task (Orientation and Shape) as a factor. For all models reported, we followed the same three-step strategy. First, we fit each model with the maximal random effects structure, including subjects’ random intercepts, within-subjects random slopes (for Switching, Difficulty, and Task), and their interactions (Switching * Difficulty). If the full random structure model did not converge, we then removed correlations between random slopes. Finally, if the resulting model still did not converge, we removed all random slopes accounting for less than 1% of the variance (Bates, Kliegl, et al., 2015; Declerck et al., 2020). This three-step strategy always resulted in convergence.

For analysis involving reaction times (RT), trials in which participants responded incorrectly were discarded (8.7% of trials). We also discarded RTs above and below 3 *SD*s of each participant’s mean within each block (6.90% of trials; 561 Stay and 645 Switch trials were discarded), resulting in 16,344 trials analyzed. We applied a log-transformation to correct for the observed skewed distributions of the data. The RT data were analyzed using linear mixed-effects regression modeling with the lmer function, including the same main factors and interactions as in the accuracy analysis and following the same model-fitting strategies.

The effects of the different predictors and their interactions on participants’ RT and accuracy were assessed through likelihood ratio tests using the car package in R (https://cran.r-project.org/web/packages/car/index.html). These tests were based on Type 3 sums of squares. Following a significant interaction, pairwise post hoc contrasts were carried out using the emmeans package in R (https://cran.r-project.org/web/packages/emmeans/index.html). All data from Experiments 1 and 2 as well as the scripts used can be found here: https://osf.io/795wx/?view_only=d15b25249a5e42cf842b65b23c88a6b6.

## EXPERIMENT 1 RESULTS

**Figure 2.**
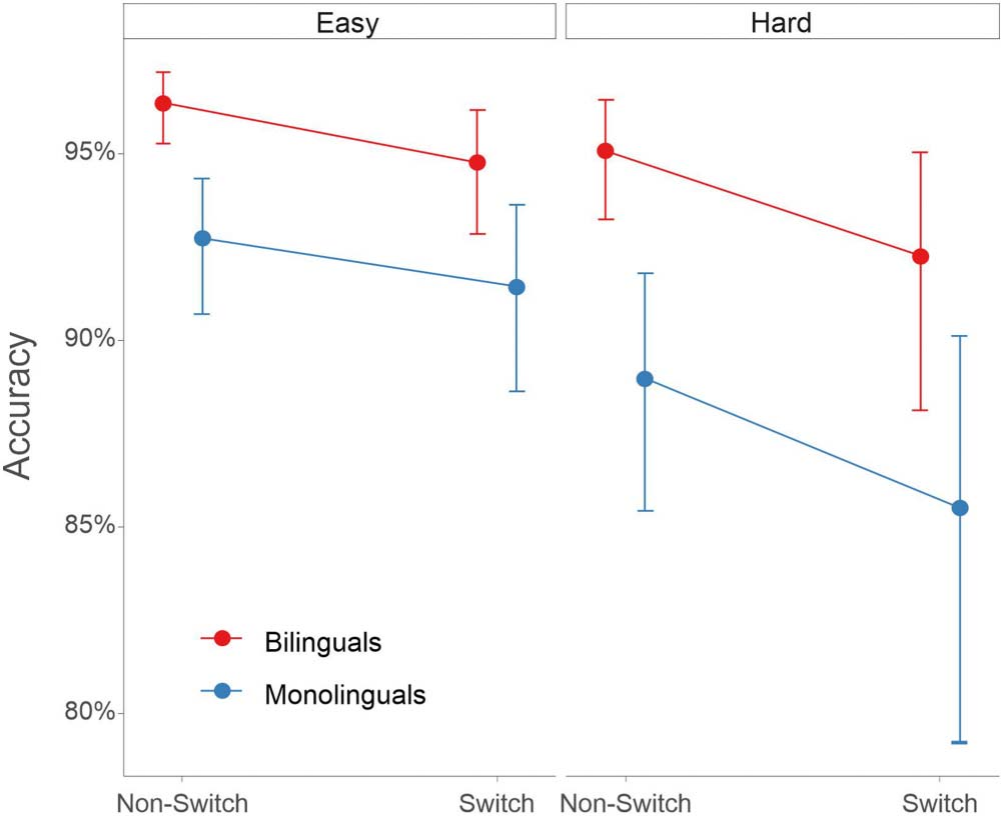
Partial effects (with 95% confidence intervals) representing the estimated accuracy for both groups in Experiment 1 under Easy and Hard conditions. Bilinguals were more accurate than monolinguals throughout the task. The plot was calculated using the ggpredict function from the ggeffects package in R (https://cran.r-project.org/web/packages/ggeffects/index.html).

Using linear mixed modeling (see Experiment 1 Materials and Methods), we investigated bilingualism’s impact on nonlinguistic task switching across different levels of cognitive demands. First, we looked at the participants’ accuracy (Figure 2). Participants were more accurate in Non-Switch than Switch trials [β = 0.44, *SE* = 0.12, χ^2^(1) = 24.76, *p* < 0.001], in Easy than Hard blocks [β = 0.36, *SE* = 0.11, χ^2^(1) = 42.91, *p* < 0.001], and performing shape than orientation judgments [β = 0.26, *SE* = 0.08, χ^2^(1) = 11.19, *p* < 0.001]. Notably, Bilinguals were more accurate than Monolinguals as revealed by a main effect of Group [β = 0.70, *SE* = 0.19, χ^2^(1) = 13.27, *p* < 0.001]. No interactions were found significant (all *p*s > 0.16).

**Figure 3.**
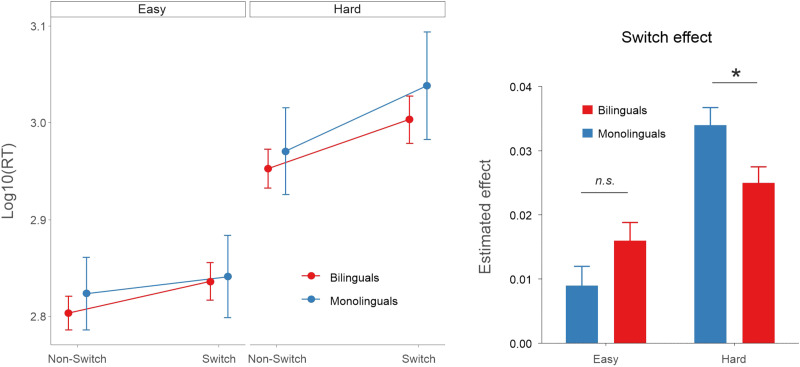
Partial effects (with 95% confidence intervals) representing the estimated reaction times (RTs; log transformation) for both groups in Experiment 1 under Easy and Hard conditions. Bilinguals presented reduced switch costs in the Hard block compared to monolinguals while no differences were found in the Easy condition. The plot was calculated using the ggpredict function from the ggeffects package in R (https://cran.r-project.org/web/packages/ggeffects/index.html).

Regarding RT (Figure 3), participants were faster in Non-Switch than Switch trials [β = 0.04, *SE* = 0.003, χ^2^(1) = 215.34, *p* < 0.001], in Easy than Hard blocks [β = 0.17, *SE* = 0.008, χ^2^(1) = 350.60, *p* < 0.001], and in shape than orientation judgments [β = 0.01, *SE* = 0.004, χ^2^(1) = 9.85, *p* = 0.002]. However, the interaction between Switching and Difficulty was significant [β = 0.03, *SE* = 0.005, χ^2^(1) = 42.21, *p* < 0.001], reflecting an increased switching cost in Hard as compared to Easy blocks. The results also revealed a significant 3-way interaction between Switching, Difficulty, and Group [β = 0.03, *SE* = 0.01, χ^2^(1) = 10.13, *p* = 0.001]. In the Easy block, switch costs were comparable between groups, although with a tendency for a larger switch cost in bilinguals than for monolinguals (*t* ratio = −1.8, *p* = 0.063). Further post hoc analysis did not reveal, however, any group effect in Stay (*t* ratio = 1.01, *p* = 0.31) or Switch trials (*t* ratio = 0.21, *p* = 0.83) in the Easy block. On the other hand, in the Hard block, bilinguals presented a reduced switch cost compared to monolinguals (*t* ratio = 2.26, *p* = 0.026), specifically due to faster RTs in Switch trials in the bilingual group (*t* ratio = 2.00, *p* = 0.049). No group differences were found in Stay trials (*t* ratio = 1.12, *p* = 0.27). These findings fit well with previous studies showing a reduced switching cost in bilinguals compared to monolinguals under the most demanding conditions.

## EXPERIMENT 2 MATERIALS AND METHODS

### Participants

One hundred and sixty-five participants were included in this study. Given that the task involves language control, bilinguals were selected depending on whether they reported speaking with both parents in Catalan and their contact with Spanish started after their L1 (sequential bilinguals, L1Cat group, *N* = 55), or whether they spoke with one parent in Catalan and with the other in Spanish, starting the contact with both languages simultaneously (simultaneous bilinguals, L1Cat/Sp group, *N* = 55). In the monolingual group participants spoke only in Spanish with both parents (*N* = 55). The three groups were equivalent in terms of age and sex distribution [L1Cat group: 45 women (mean age = 20.6, *SD* = 2.4); L1Cat/Sp group: 44 women (mean age = 20.6, *SD* = 3.1); monolingual group: (40 women, mean age = 21, *SD* = 3.6]. As in Experiment 1, both groups of bilingual participants completed the LHQ and the BSWQ.

Although both groups of bilinguals were highly proficient in both languages (values higher than 3.5 on a scale of 4, see Table 2), they differed in their own perceived proficiency level in Catalan and Spanish. The L1Cat group reported feeling more proficient in Catalan than in Spanish [*t*(48) = −5.6, *p* < 0.001] while L1Cat/Sp bilinguals reported feeling equally proficient in both [*t*(48) = 1.3, *p* = 0.19]. In addition, both groups showed comparable scores in the contextual [*t*(94) = 1.18, *p* = 0.24] and unattended facets [*t*(94) = 0.99, *p* = 0.99] of the BSWQ. However, the L1Cat group tended to switch more from Spanish to Catalan than the L1Cat/Sp group [*t*(94) = 2.59, *p* = 0.011], while the latter tended to switch more from Catalan to Spanish than the L1Cat group [*t*(94) = 2.28, *p* = 0.025], consistent with their language dominance. Besides, when considering both groups of bilinguals together (L1Cat/Sp and L1Cat), they showed similar LHQ [Language dominance: *t*(141) = 1.70, *p* = 0.09; Proficiency in Spanish: *t*(133) = 1.16, *p* = 0.25; Proficiency in Catalan: *t*(133) = 1.34, *p* = 0.18] and BSWQ scores [Spanish Switch: *t*(133) = 0.30, *p* = 0.77; Catalan Switch: *t*(133) = 1.65, *p* = 0.10; Contextual Switch: *t*(133) = 0.89, *p* = 0.38; Unattended Switch *t*(133) = 0.72, *p* = 0.47; Overall Switch: *t*(133) = 0.64, *p* = 0.52] compared to the bilingual group of Experiment 1. They were also comparable in terms of age and gender (*p*s > 0.8), and were students of the same university as the bilingual group of Experiment 1. All groups from Experiment 2, including monolinguals, were equivalent in terms of verbal IQ as measured by the vocabulary subscale from the verbal scale of the Wechsler Adult Intelligence Scale—Third Edition (WAIS-III; Wechsler, 1997) (*F* < 1; see Table 2).

**Table 2.** Language history, self-evaluated proficiency scores, and BSWQ scores for the two bilingual groups in Experiment 2.

	**L1Cat**		**L1Cat/Sp**		**Monolinguals**
*N*	48		47		46
Age	20.69 (0.34)		20.55 (0.45)		21.00 (0.53)
Vocabulary (WAIS-III)	44.10 (0.66)		44.15 (0.60)		43.76 (0.77)
*Language History Questionnaire*					
Use of Language	1.95 (0.07)		3.84 (0.09)		
*Proficiency scores*	*Spanish*	*Catalan*	*Spanish*	*Catalan*	
Age of acquisition	3.9 (0.2)	2.2 (0.07)	2.4 (0.1)	2.5 (0.12)	
Comprehension	3.85 (0.05)	3.96 (0.03)	3.98 (0.02)	4 (0)	
Reading	3.54 (0.08)	3.79 (0.07)	3.85 (0.05)	3.72 (0.07)	
Production	3.90 (0.04)	3.98 (0.02)	4 (0)	4 (0)	
Writing	3.31 (0.09)	3.90 (0.04)	3.83 (0.06)	3.74 (0.06)	
Total	3.65 (0.05)	3.91 (0.03)	3.91 (0.03)	3.87 (0.03)	
*BSWQ scores*					
Sp_Cat	6.43 (0.32)		8.77 (0.37)		
Cat_Sp	9.87 (0.28)		8.79 (0.32)		
Contextual	7.44 (0.35)		7.85 (0.43)		
Unattended	6.70 (0.40)		7.44 (0.45)		
Overall	30.45 (0.92)		32.85 (1.15)		

*Note*. Age and scores are reported as mean (standard error of the mean). BSWQ = Bilingual Switching Questionnaire. WAIS-III = Wechsler Adult Intelligence Scale (Wechsler, 1997). Sp_Cat = Tendencies to switch from Catalan to Spanish; Cat_Sp = Tendencies to switch from Spanish to Catalan; L1Cat = sequential Catalan dominant bilinguals; L1Cat/Sp = simultaneous balanced bilinguals.

All participants had normal or corrected-to-normal vision. Seven participants from the L1Cat group, eight from the L1Cat/Sp, and nine from the monolingual group were not included in the analysis because they had error rates larger than 30% in the experimental task. One participant from the L1Cat and one from the L1Cat/Sp did not complete the full experiment due to technical problems but were included in the analysis since they completed >85% of the task (completing 199 and 202 trials, respectively, out of 224). As in Experiment 1, all volunteers were university students. Bilinguals were born and tested in Catalonia, and monolinguals were born and tested in Andalusia. As in Experiment 1, only participants reporting a low frequency of English use (or other languages) were selected for the study. All monolingual participants self-labeled as monolinguals. They were paid cash or course credits for their participation, and they all signed a consent form approved by the local ethics committee.

### Stimuli and Procedure

**Figure 4.**
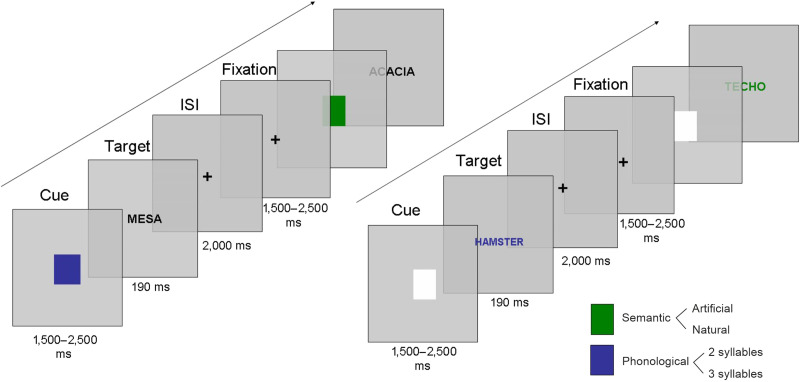
Sequence of events in the two types of blocks used in Experiment 2. Easy block included colored squares with words in black typeface (left panel), and the Hard block included white squares and words in colored typeface (right panel).

We used the same switching paradigm as in Experiment 1 but including linguistic stimuli instead of geometric shapes, and using green or blue for the color of the cues (Figure 4). Based on the color cue, participants were asked to perform either phonological (decide whether a word had two or three syllables) or semantic judgments (decide whether a word represented something natural or human-made) for upcoming Spanish target words. The task was divided into two blocks (Easy and Hard) as in Experiment 1.

We used two lists of 56 concrete words as targets. Words were matched between lists in number of letters (*M* = 5.7, *SD* = 0.7) and surface frequency (*M* = 26.5, *SD* = 5.2) (Alameda & Cuetos, 1995). Half of the words in each list represented natural items, and the other half a set of artificial items or human-made objects. In each set, half of the stimuli were bisyllabic words, and the other half were trisyllabic. Half of the words were cognates in each of these sets, and the other half were non-cognates.

As in Experiment 1, a total of 112 different stimuli were presented twice, once per block (Easy, Hard). Words were presented in black Arial 22-point typeface inside a grey 3.5 cm × 1.25 cm box (which matched the background color of the display).

### Statistical Analysis

The same analyses as in Experiment 1 were performed but with three language groups (L1Cat, L1Cat/Sp, and Monolinguals) and Cognate status (cognates and non-cognates) as a fifth factor. Across all included participants, 31,538 trials were collected. From those, 26,783 trials (85%) were included in the final Accuracy analysis. For the RT analysis, (i) incorrect trials (8.7% of trials) and (ii) RTs above and below 3 *SD* of each participant’s mean within each block (6.90% of trials, 621 Stay and 673 Switch trials) were discarded, resulting in 21,956 trials being finally included.

A big model, including both experiments, was also built to investigate potential interactions between experiments, including the 4-way interaction between Switching, Difficulty, Group, and Experiment. Finally, in this last analysis, the two groups of bilinguals of Experiment 2 (L1Cat and L1Cat/Sp) were merged into one bilingual group.

## EXPERIMENT 2 RESULTS

**Figure 5.**
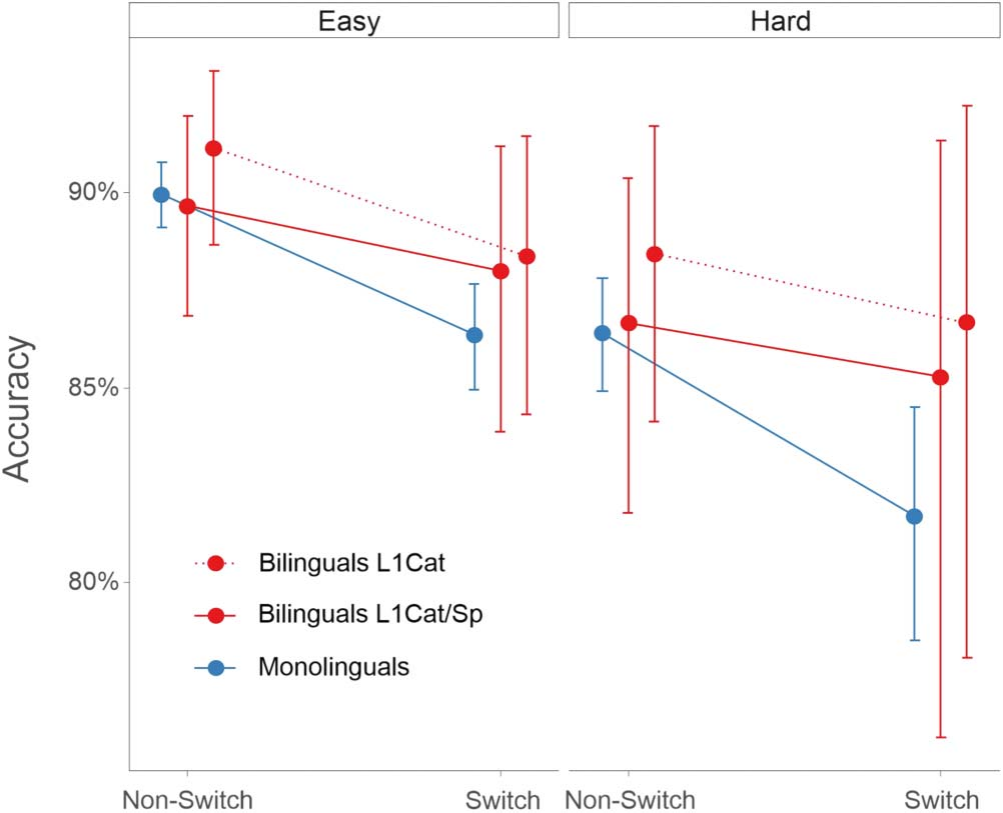
Partial effects (with 95% confidence intervals) representing the estimated accuracy for the three groups in Experiment 2 under Easy and Hard conditions. No differences among groups were found significant. The plot was calculated using the ggpredict function from the ggeffects package in R (https://cran.r-project.org/web/packages/ggeffects/index.html). L1Cat = sequential Catalan dominant bilinguals; L1Cat/Sp = simultaneous balanced bilinguals.

As in Experiment 1, we implemented linear mixed modeling (see Experiment 2 Materials and Methods) to investigate bilingualism’s impact on a verbal task-switching paradigm. Concerning accuracy rates, participants were more accurate in Non-switch than Switch trials [β = 0.24, *SE* = 0.04, χ^2^(1) = 42.73, *p* < 0.001], and in the Easy than Hard block [β = 0.28, *SE* = 0.04, χ^2^(1) = 42.16, *p* < 0.001]. Crucially, no main effect of Group or interactions including Group were found significant (all *ps* > 0.06; Figure 5).

**Figure 6.**
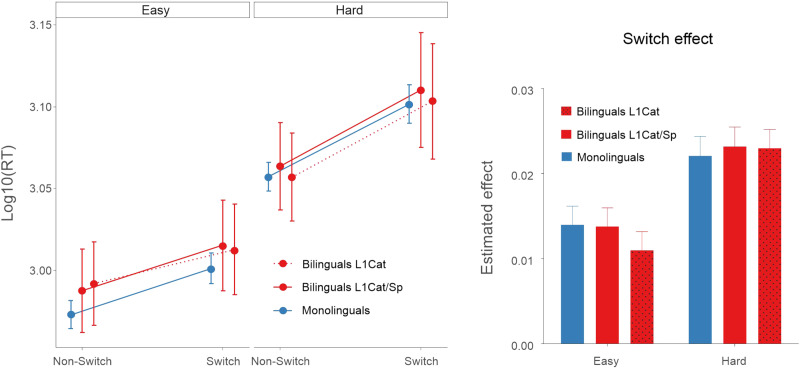
Partial effects (with 95% confidence intervals) representing the estimated reaction times (RTs; log transformation) for the three groups in Experiment 2 under Easy and Hard conditions. No differences among groups were found significant. The plot was calculated using the ggpredict function from the ggeffects package in R (https://cran.r-project.org/web/packages/ggeffects/index.html). L1Cat = sequential Catalan dominant bilinguals; L1Cat/Sp = simultaneous balanced bilinguals.

RT analysis revealed that participants were faster in Non-Switch than in Switch trials [β = 0.04, *SE* = 0.002, χ^2^(1) = 343.02, *p* < 0.001], and in the Easy than the Hard block [β = 0.09, *SE* = 0.004, χ^2^(1) = 389.91, *p* < 0.001]. In addition, participants presented larger switching costs in the Hard than the Easy block [Switching * Difficulty: β = 0.02, *SE* = 0.004, χ^2^(1) = 32.79, *p* < 0.001]. No other main effects or interactions were found significant (all *p*s > 0.21). Thus, no bilingual advantage was present in Experiment 2. (See Figure 6.)

To compare the effects observed in the two experiments, we ran a model with both experiments, including the 4-way interaction between Switching, Difficulty, Group, and Experiment. Concerning accuracy, the interaction between Group and Experiment was found significant [β = 0.53, *SE* = 0.19, χ^2^(1) = 7.57, *p* = 0.006]. The bilingual advantage on accuracy from Experiment 1 (*z* ratio = 4.69, *p* < 0.001) was greater than in Experiment 2, where no bilingual advantage existed (*z* ratio = 1.29, *p* = 0.20). Finally, a 3-way interaction between Switching, Group, and Experiment was found significant [β = 0.40, *SE* = 0.14, χ^2^(1) = 7.71, *p* = 0.005]. This interaction revealed that the Group × Experiment interaction previously described was greater for Stay (*z* ratio = 3.49, *p* < 0.005) than Switch trials (*z* ratio = 1.64, *p* = 0.10).

Regarding RT, the 4-way interaction between Switching, Difficulty, Experiment, and Group was found significant [β = 0.04, *SE* = 0.01, χ^2^(1) = 10.61, *p* < 0.001]. Bilinguals showed a smaller switching cost than monolinguals in the Hard condition only in Experiment 1 (*t* ratio = 2.86, *p* = 0.005).

Finally, we also examined whether language dominance, as measured by the LHQ (see Experiment 2 Materials and Methods), influenced bilinguals’ performance. We ran a model including the 4-way interaction between Switching, Difficulty, Language dominance, and Experiment for bilinguals’ accuracy and RT data. Regarding accuracy, no main effect of language dominance nor any interaction with other factors was found significant (all *p*s > 0.10). Similar findings were found with the RT data, with no main effect of language dominance or interaction between language dominance and any other factor (all *p*s > 0.09).

## DISCUSSION

The main goal of the current study was to investigate whether the bilinguals’ engagement of executive functions to manage two languages hinders the potential bilingual advantage in task-switching functions when shifting between tasks involving language processing. A large body of research has specifically investigated the impact of bilingualism on nonlinguistic task-switching functions, offering contradictory results (Barac & Bialystok, 2012; Brito et al., 2016; Crivello et al., 2016; de Bruin et al., 2015; Garbin et al., 2010; Gold et al., 2013; Hartanto & Yang, 2019; Hernández et al., 2013; Houtzager et al., 2017; Kuipers & Westphal, 2021; Marzecová et al., 2013; Mor et al., 2015; Moradzadeh et al., 2015; Paap & Greenberg, 2013; Paap & Sawi, 2014; Poulin-Dubois et al., 2011; Prior & Gollan, 2011; Prior & MacWhinney, 2010; Rodríguez-Pujadas et al., 2013; Shulley & Shake, 2016; Stasenko et al., 2017; Wiseheart et al., 2016; Woumans et al., 2019). Nevertheless, recent meta-analyses indicate that, although much smaller than initially thought, there is a certain bilingual advantage in nonlinguistic task-switching paradigms (Gunnerud et al., 2020; Ware et al., 2020). In agreement with this hypothesis, our results indicate that bilinguals presented reduced switch costs and were more accurate in a nonlinguistic discrimination task.

The differences in switch-cost magnitudes were due to faster responses of bilinguals in switch trials. Notably, this effect was confined to the most demanding condition, in which participants could not prepare in advance (Monsell, 2003). These findings align with previous reports showing that the bilingual advantage is generally observed only in the most demanding situations (Bialystok et al., 2006; Costa et al., 2009; Hernández et al., 2013). A recent study (Kuipers & Westphal, 2021) has provided further evidence in favor of this hypothesis by manipulating task complexity, just as was done in the present study. The study manipulated the temporal interval between the cue and the target and observed a bilingual advantage in executive functions only in those conditions that were more demanding (i.e., when participants had less time to prepare).

In addition, bilinguals were overall more accurate in the nonlinguistic task switching paradigm than monolinguals. While similar overall effect on bilinguals’ performance in executive function tasks has been observed in some studies (Hartanto & Yang, 2019; Soveri et al., 2011), others have restricted this effect to a larger domain-general global accuracy (Sanchez-Azanza et al., 2020). This bilinguals’ advantage has been suggested to reveal their ability to manage tasks that involve mixing trials of different types, which would reflect better monitoring processes in this group as a consequence of the need to constantly evaluate the language to be used in each communicative context (Barac & Bialystok, 2012; Costa et al., 2009). Taken together, our results suggest that bilingualism may boost both monitoring and switching abilities in nonlinguistic task-switching paradigms.

The bilingual advantage in nonlinguistic task-switching paradigms fits well with the idea that bilinguals’ cognitive benefits are more likely to appear on tasks similar to bilingual language use, such as switching tasks, rather than inhibition or working memory tasks. The competition between two tasks in task-switching paradigms may be comparable to the continuous activation and competition of two different languages in bilinguals. Indeed, the brain circuits underlying language control and nonlinguistic task switching partially overlap. Neuroimaging studies have revealed a cortico-subcortical network implicated in language control that overlaps with the domain-general executive control network (ECN), including the anterior cingulate cortex, inferior frontal cortex, basal ganglia, and parietal cortices (Abutalebi & Green, 2007, 2016; Baumgart & Billick, 2018; De Baene et al., 2015; Garbin et al., 2010; Rodriguez-Fornells et al., 2006; van Heuven et al., 2008; Weissberger et al., 2015). Most of these regions are not typically involved in monolingual language processing (e.g., Friederici, 2011). Neuroplasticity changes in the bilingual brain due to the extra training that language control represents may lead them to recruit the ECN more efficiently than matched monolinguals (Abutalebi et al., 2012), causing behavioral advantages on cognitive control tasks (Del Maschio et al., 2018; Timmer et al., 2019). On the other hand, neuroimaging studies have shown that bilinguals, compared to monolinguals, recruit brain regions involved in language control when performing nonlinguistic cognitive tasks, particularly in task-switching paradigms (Garbin et al., 2010; Rodríguez-Pujadas et al., 2013). However, the additional recruitment of language control brain circuits do not always turn into a benefit when bilinguals perform nonlinguistic cognitive tasks. For instance, transcranial direct current stimulation over the left dorsolateral prefrontal cortex (DLPFC), considered to be crucial for language control (Abutalebi & Green, 2016; Klaus & Schutter, 2018), impairs nonlinguistic task switching but aids language control in bilinguals (Vaughn et al., 2021). This may suggest that, although language and cognitive control rely on partially overlapping brain areas, the implication of specific ECN regions such as the left DLPFC is opposite when it comes to cognitive or language control.

Therefore, could the bilingual advantage source lead to a cost when performing task switching demanding language control? Although bilinguals are remarkably efficient in controlling interference from the non-target language, tasks involving language control may become more demanding for bilinguals than monolinguals. Our findings do support this hypothesis. Concretely, we show that the bilingual advantage observed in nonlinguistic task switching vanishes when language is present. Notably, the lack of group effects in the within-language task-switching paradigm could not be attributed to worse overall performance in bilinguals than monolinguals and, therefore, due to slower lexical access and processing of the Spanish language in bilinguals (Bialystok et al., 2009). First, both groups presented comparable accuracy and RTs throughout the task. Second, bilingual and monolingual participants did not differ in their verbal abilities (evaluated by WAIS-III in Spanish). Finally, all bilingual groups reported high levels of proficiency in both languages.

Previous studies investigating the bilingual advantage in task switching have also used linguistic material (Brito et al., 2016; Brown, 2015). In both studies, participants had to switch between classifying objects and words. However, the authors did not investigate any potential effect of task or interaction between task performance and differences in language dominance among bilinguals. In addition, Prior and Gollan (2013) used a color-shape switching task in which participants produced verbal responses in their dominant language. Notably, none of them found a bilingual advantage in switching costs. Our findings thus provide a new perspective to interpret these null effects. That is, the additional cognitive demands imposed on bilinguals when dealing with tasks involving language control make their performance comparable to that of monolinguals. Our findings also speak of the bilingual advantage’s fragility, which is only observable in high demanding conditions that do not require linguistic processing.

However, contrary to our initial hypothesis, we did not find any effect of language dominance or use. Similar neural mechanisms seem to underlie L1 and L2 processing in the bilingual brain (Abutalebi, 2008; Perani et al., 1998). However, L2 tends to show a more extended pattern of activations than L1, which depends on the level of proficiency in L2 and likely reflects the more effortful processing of the second language to avoid interference from L1, mainly when there is a strongly dominant L1 (Abutalebi, 2008). We, therefore, hypothesized that individuals with strong Catalan dominance would require more resources than Spanish dominant bilinguals to avoid interference from the non-target language (Catalan) during task performance. The lack of behavioral differences between Catalan and Spanish-dominant bilinguals and the absence of significant correlations with language use measures indicate that language dominance did not incur any cost or benefit in the tested population. However, it is important to note the high proficiency levels and use of both languages in the bilingual population tested. The inclusion of different bilingual groups with larger language dominance and use differences may be more convenient to test for potential language dominance effects, avoiding any ceiling effect due to high proficiency and use.

### Limitations

The current study also presents several limitations that need to be considered. One limitation is the between-participants design employed. However, Monolinguals and Bilinguals from Experiments 1 and 2 included students from the same university (University of Barcelona and University of Granada, respectively), of similar age and with similar gender proportion, and, therefore, were comparable across experiments. In addition, the language backgrounds of the different groups of bilinguals were comparable as well. A second limitation is the lack of control for socioeconomic differences between groups, particularly considering that bilinguals and monolinguals were recruited from different geographic areas, although, as stated earlier, they were all university students of similar age and gender (from Catalonia and Andalusia, respectively). Previous studies have shown that socioeconomic status (SES) correlates with measures of cognitive performance and language ability throughout development, with higher SES associated with better outcomes (Bradley & Corwyn, 2002; Sirin, 2005). However, it is important to note that our findings revealed not only a main effect of bilingualism in the nonlinguistic task-switching paradigm but also differences between the Spanish/Catalan bilinguals from Experiments 1 and 2 as a function of the type of task performed. Notably, these two groups of bilinguals were recruited from the same university and showed similar proficiency and language use. Therefore, we find it very unlikely that the main findings reported here—an interaction between bilingualism and language control in task switching—are driven by uncontrolled SES differences or our between-participants design. However, we acknowledge that further studies involving within-participant designs and controlling for SES may provide important insights into the role of language control and SES in executive function skills.

A third limitation to consider is the different nature and difficulty of the tasks used in both experiments despite sharing the same task schema and timing. Indeed, participants in Experiment 1 (nonlinguistic switching task) were faster and more accurate than participants in Experiment 2 (within-language switching task), regardless of the language group they belonged to (Bilinguals or Monolinguals). However, we believe this difference in task difficulty plays in favor of our conclusions. As previously stated, the bilingual advantage is generally found in highly demanding scenarios, and therefore, we would expect an even greater bilingual advantage in the within-language task. Yet, this was not the case, rather the opposite. However, further studies with non- and within-language task switching paradigms of similar difficulty may help disentangle the contribution of task difficulty to our findings and the potential interactions between task difficulty and the bilingual advantage in executive functions.

### Conclusions

In sum, the present study suggests that the heightened levels of training that bilinguals have on dealing with two languages during their daily lives benefit their task-switching skills compared to monolinguals, but only when the task imposes greater executive demands and does not involve language control. When the tasks require participants to perform judgments on words, the bilinguals’ reduction in switching costs observed with nonlinguistic material vanishes, and bilinguals' and monolinguals’ performance is equated. This is the first behavioral evidence that language control demands may limit the bilingual advantage in general-domain executive functions. These findings have important implications and open new avenues of research on investigating the bilingual mind and its potential benefits on executive functions.

## ACKNOWLEDGMENTS

This work was supported by the BFU2017-87109-P Grant from the Spanish Ministerio de Ciencia e Innovación (Ruth de Diego-Balaguer), which is part of Agencia Estatal de Investigación (AEI) (co-funded by the European Regional Development Fund [ERDF]—a way to build Europe) and CERCA Programme / Generalitat de Catalunya for institutional support to IDIBELL. Ernest Mas-Herrero received support of “la Caixa” Foundation (ID 100010434) and from the European Union’s Horizon 2020 research and innovation programme under the Marie Skłodowska-Curie grant agreement No 847648 (LCF/BQ/PI20/11760001).

## FUNDING INFORMATION

Ruth de Diego-Balaguer, Ministerio de Ciencia e innovación, Award ID: BFU2017-87109-P. Ernest Mas-Herrero, “la Caixa” Foundation (https://dx.doi.org/10.13039/100010434), Award ID: LCF/BQ/PI20/11760001.

## AUTHOR CONTRIBUTIONS

**Ernest Mas-Herrero**: Conceptualization: Supporting; Formal analysis: Lead; Writing – original draft: Equal; Writing – review & editing: Equal. **Daniel Adrover-Roig**: Formal analysis: Supporting; Writing – original draft: Supporting; Writing – review & editing: Supporting. **María Ruz**: Conceptualization: Equal; Formal analysis: Supporting; Methodology: Equal; Supervision: Supporting; Writing – original draft: Supporting; Writing – review & editing: Supporting. **Ruth de Diego-Balaguer**: Conceptualization: Equal; Formal analysis: Supporting; Supervision: Lead; Writing – original draft: Equal; Writing – review & editing: Equal.
